# Two Rare Cases of Bilateral Diaphragmatic Paralysis in Neonates

**DOI:** 10.3390/pediatric17060127

**Published:** 2025-12-01

**Authors:** Sara Ronci, Chiara Maddaloni, Stefano Caoci, Stefano Pro, Daniela Longo, Andrea Conforti, Andrea Dotta, Francesca Campi

**Affiliations:** 1 Neonatal Intensive Care Unit, “Bambino Gesù” Children’s Hospital IRCCS, 00165 Rome, Italy; chiara.maddaloni@opbg.net (C.M.); stefano.caoci@opbg.net (S.C.); andrea.dotta@opbg.net (A.D.); francesca.campi@opbg.net (F.C.); 2 Developmental Neurology Unit, “Bambino Gesù” Children’s Hospital IRCCS, 00165 Rome, Italy; stefano.pro@opbg.net; 3 Functional and Interventional Neuroradiology Unit, “Bambino Gesù” Children’s Hospital IRCCS, 00165 Rome, Italy; daniela.longo@opbg.net; 4 Neonatal Surgery Unit, Area of Fetal, Neonatal and Cardiological Sciences, “Bambino Gesù” Children’s Hospital IRCCS, 00165 Rome, Italy; andrea.conforti@opbg.net

**Keywords:** bilateral diaphragmatic paralysis, respiratory failure, diaphragmatic plication

## Abstract

Diaphragmatic paralysis (DP) in neonates is a rare yet potentially life-threatening cause of respiratory distress, often resulting from obstetric trauma or cardiac surgery. This report presents two distinct cases of bilateral DP: one following a dystocic delivery with associated brachial plexus involvement, and the other linked to a genetic mutation (SYNGAP1) in a neonate with no birth trauma. Diagnosis was established through imaging, fluoroscopy, electromyography, and genetic testing. In both cases, conservative management was initially pursued; however, due to persistent respiratory failure, invasive interventions were required. The first patient underwent bilateral diaphragmatic plication with favorable outcomes, while the second required tracheostomy due to poor response to non-invasive ventilation with good outcome. These cases highlight the diagnostic and therapeutic challenges of neonatal DP, emphasizing the need for individualized treatment strategies in the absence of standardized guidelines. Early diagnosis and a multidisciplinary approach are crucial to optimize respiratory outcomes and reduce complications from prolonged mechanical ventilation.

## 1. Introduction

Diaphragmatic paralysis (DP) in neonates is a rare cause of respiratory distress and it can be a life-threatening situation [1,2]. The main causes of DP in newborns are obstetric trauma at birth and sequelae of cardiac surgery [3,4].

The prevalence of DP following cardiac surgery is reported to be between 0.3% to 12.8% and mostly affects Blalock–Taussig shunt procedures, patch correction of ventricular septal defect and surgery for transposition of the great vessels [5].

The incidence of DP following obstetric trauma is reported to be between 1/15,000 and 1/30,000 live births [6]. The involvement is usually unilateral, most often affecting the right hemidiaphragm [7]. This condition should be suspected in cases of respiratory distress following a difficult delivery [1]. These newborns may also present with perinatal asphyxia, humerus and clavicle fractures, and ipsilateral brachial plexus paralysis with motor deficits in the upper limb [8]. The latter occurrence is explained by the anatomical proximity of the phrenic nerve (PN) roots (C3 to C5) and the brachial plexus (BP) (C5 to T1) [9].

Due to the mechanical nature of the damage, which involves the stretching of the nerve roots during the extraction maneuver, only rare anecdotal cases of bilateral DP following dystocic delivery are reported in the literature. Most frequently, unilateral PD is described with associated ipsilateral brachial plexus palsy (BPP) [2,10,11,12].

Finally, neuromuscular disorders are rare causes of diaphragmatic paralysis [13].

Case reports describe cases of diaphragmatic paralysis associated with spinal muscular atrophy [14], myasthenia gravis [15], and congenital myopathies [16]. Bosman et al. also report a case of diaphragmatic paralysis where the histologic findings showed a dystrophy-like muscle pathology restricted to the diaphragm resulting in partial diaphragmatic hypoplasia [17].

Bedside ultrasound will gradually replace fluoroscopy, which is still the gold standard for DP confirmation. To better characterize phrenic nerve injury and recovery chances, some electrophysiological methods might be helpful [9]. Currently, there is no established therapeutic protocol in the literature for the management of DP. Depending on the duration and severity of symptoms, treatment may range from a wait-and see approach to non-invasive or invasive respiratory support and even surgery.

**Figure 1:**
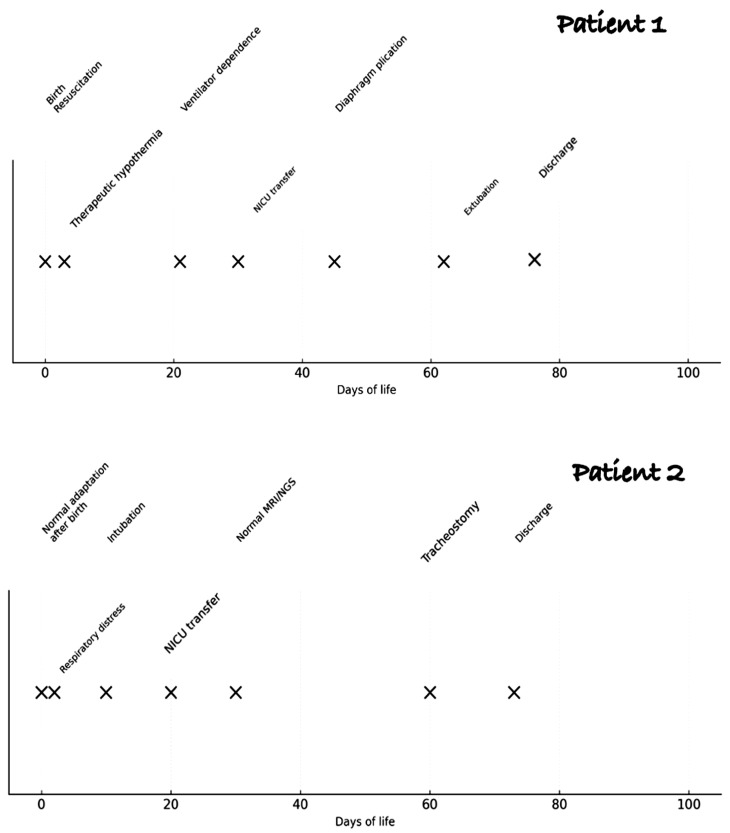
Timeline of key events from birth to discharge of both patients.

We present two cases of bilateral diaphragmatic paralysis, each characterized by distinct features: the first, resulting from a dystocic delivery without additional neurological complications, and the second, linked to a mutation in the SYNGAP1 gene associated with autosomal dominant type 5 neurodevelopmental disorder. We also outline the two different treatments administered to our patients, both leading to successful discharge without the need for ventilatory support (Table 1). A timeline of the hospitalization of both patients is described in Figure 1.

## 2. Case Reports

***Patient 1***

The first patient is a male term newborn, born at 37 + 5 weeks of gestation via operative delivery due to shoulder dystocia. At birth, the infant was apneic with a heart rate below 100 bpm, requiring immediate resuscitation, including intubation and mechanical ventilation. His Apgar scores were 0, 3, and 6 at 1, 5, and 10 min, respectively. A left humerus fracture with hypomobility of the left arm was observed during the initial examination. Following national guidelines for perinatal asphyxia, he received therapeutic hypothermia for 72 h.

Despite multiple attempts to extubate him, the infant remained dependent on mechanical ventilation. At three weeks of age, fluoroscopic assessment of diaphragm mobility revealed inadequate bilateral diaphragm excursion. At one month of age, the infant was transferred to our neonatal intensive care unit (NICU) for further management. Upon arrival, he was in critical condition, intubated, and mechanically ventilated. Neurological examination showed persistent hypomobility of the left arm, which remained in adduction and internal rotation, consistent with BP involvement.

**Figure 2:**
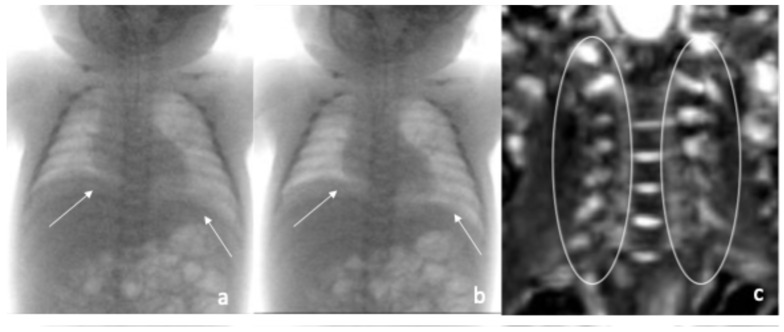
(**a**): Fluoroscopic evaluation of diaphragm in expiratory phase: the white arrows show the poor diaphragmatic excursion in the two respiratory phases. (**b**): Fluoroscopic evaluation of diaphragm in inspiratory phase: the white arrows show the poor diaphragmatic excursion in the two respiratory phases. (**c**): Magnetic Resonance Imaging of the brachial plexus: the white circles show the roots of the brachial plexus with no sign of avulsion.

Fluoroscopic evaluation of diaphragm mobility was performed both under mechanical ventilation and spontaneous breathing. During assisted ventilation, symmetrical diaphragm excursion was noted to the seventh intercostal space. After ventilator disconnection, the diaphragm ascended to the sixth intercostal space with abdominal breathing, but no movement was detected (Figure 2a,b).

X-rays confirmed a fracture in the middle third of the left humeral diaphysis with distanced stumps and a robust healing bone callus. CMAP analysis of the PN showed bilateral activity, with reduced left-sided response. EMG revealed moderate peripheral neurogenic injury, affecting the C5–C6 roots on the right side, with signs of reinnervation but no active denervation. MRI of the BP revealed normal root courses bilaterally, with no signs of avulsions (Figure 2c).

After multidisciplinary consultation, and in the absence of a clear neuromuscular origin, the decision was made to proceed with bilateral diaphragmatic plication at 45 days of age. The diaphragmatic plication was performed by pediatric surgeons through a median supraumbilical laparotomy. During surgery, diaphragm biopsies demonstrated normal-sized striated muscle fibers with preserved vascularization and no inflammatory signs.

Post-surgery, the infant was successfully extubated at 62 days of age, initially supported by non- invasive ventilation and later transitioning to spontaneous breathing without complications. A brain MRI, conducted as part of the protocol for perinatal asphyxia at 2 months of age, showed no significant abnormalities in the supra- and subtentorial regions. Additionally, electroencephalogram (EEG) findings were normal. He was discharged at 76 days of life.

To date, this case represents the first documented instance of bilateral diaphragmatic paralysis without other associated nerve damage, apart from moderate right brachial plexus impairment due to the dystocic delivery. Genetic studies, including exome sequencing, were negative.

At the 1-year follow-up, developmental assessment using the Bayley III scales showed normal cognitive (90) and language (89) development, although motor skills were delayed (73). Parents also reported motor difficulties in daily activities. At the 2-year follow-up, the infant showed normal brain MRI findings and neurological evaluation, demonstrating good social interaction with some attentional issues, but no localized deficiencies. He is currently undergoing physiotherapy and speech rehabilitation.

***Patient 2***

The second patient is a male term neonate, large for gestational age (LGA), born in another hospital via vaginal delivery at 40 weeks + 1 day of gestation with a birthweight of 4200 g. The pregnancy occurred spontaneously and progressed normally. Good cardio-respiratory adaptation, APGAR score of 10 at 5 min. On physical examination, the patient was macrosomic, macrocephalic, with no facial dysmorphia. He presented with symmetrical motor activity in all four limbs, normal facial mimicry and axial tone, and normal osteotendinous reflexes.

On the second day of life, he developed tachypnea and respiratory distress, for which respiratory assistance was started, first with High Flow Nasal Cannula (HFNC) and subsequently with nasal Continuous Positive Airway Pressure (nCPAP). Chest X-ray and lung ultrasound (LUS) showed increased lung interstitial tissue, consolidations in the apical regions bilaterally and in the right lower lobe. Empiric antibiotic therapy was also started.

**Figure 3:**
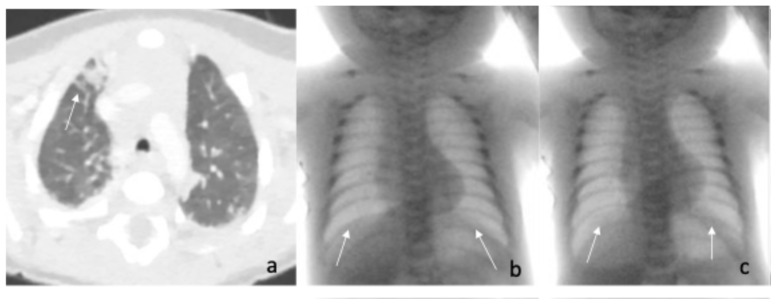
(**a**): Computed tomography angiography of the pulmonary arteries: the white arrow indicates an area of consolidation. (**b**,**c**): Expiratory (on the left side) and inspiratory (on the right side) fluoroscopy sequences. The white arrows show the poor diaphragmatic excursion in the two respiratory phases.

Due to worsening of the respiratory failure, the newborn was intubated and placed on conventional mechanical ventilation on the 10th day of life. Surfactant protein and alpha-1 antitrypsin levels were measured and found to be within normal ranges. A fibro laryngoscopy was negative, and a Computed tomography angiography of the pulmonary arteries confirmed the areas of consolidation seen on the X-ray and excluded vascular anomalies (Figure 3a).

On the 20th day of life, he was transferred to our NICU, where a fluoroscopic examination of the diaphragm showed reduced diaphragmatic excursion, approximately one intercostal space bilaterally, with abdominal-type breathing (Figure 3b,c). Electroneuromyography of the diaphragm showed no abnormalities, and given the normal neurological examination, neuromuscular causes for the diaphragmatic hypomobility were excluded. The EEG was within normal limits.

MRI of the brain and spinal cord, performed at 30 days of life, did not show hypoxic lesions or structural abnormalities. Additionally, a Next Generation Sequency (NGS) panel for neonatal respiratory failure was performed, which also came back normal.

For the evaluation of macrosomia, cortisol, insulin, IGF1, IGFBP3, GH, and thyroid profile tests were conducted, all of which were within normal ranges. Several attempts were made to wean him off mechanical ventilation, but all failed due to worsening respiratory distress and hypercapnia.

Thus, at 2 months of life, a tracheostomy was performed, after which the patient was successfully weaned off mechanical ventilation, allowing for discharge with spontaneous breathing through the tracheostomy tube on the 73rd day of life. A repeat fluoroscopic study of the diaphragm before discharge showed no improvement compared to the previous study.

Single nucleotide polymorphism-array (SNP-array) study and exome sequencing were also performed, revealing paternal UPD of chromosome 20, a genetic condition associated with pseudohypoparathyroidism type 1B (OMIM #603233) and a de novo heterozygous missense variant c.2983C>T in the SYNGAP1 gene, which causes the amino acid change p.Pro995Ser (rs1554122279) at the protein level. Pathogenic variants in the SYNGAP1 gene are associated with autosomal dominant neurodevelopmental disorder type 5 (OMIM: #612621).

A recent Chinese case series from 2022 describes 10 patients with SYNGAP1 mutation. All had motor delay and intellectual disability; 4 patients had seizures and one of these had severe hypotonia [18].

Although neither of these two mutations can explain the diaphragmatic hypomobility, it is possible that the complete loss of heterozygosity of chromosome 20 increases the risk of recessive diseases associated with genes on this chromosome, potentially involving gene segments with currently unknown expression.

Further tests were performed to study bone metabolism, revealing elevated PTH levels (67.2 pg/mL), with normal levels of calcium, phosphorus, ALP, and vitamin D. Additionally, we can also hypothesize that hormonal deregulation related to the genetic mutation could have a role in hypotonia and muscle weakness, even with normal levels of calcium and phosphorus.

The patient was assessed from a pneumological standpoint at the age of two at the last follow-up, and it was recommended that the tracheostomy be kept closed during the day. A neurological evaluation 24 months of age revealed mild psychomotor delay and clumsiness in walking, for which physiotherapy will continue.

## 3. Discussion

DP is a rare cause of neonatal respiratory distress in neonates [1]. The clinical presentation can vary, ranging from mild tachypnea with asymmetric chest excursions in unilateral forms to paradoxical breathing, cyanosis, and respiratory failure with inability to wean from mechanical ventilation in rare bilateral forms [6]. Extra-respiratory manifestations such as feeding difficulties, gastroesophageal reflux, or vomiting may also be present [8].

### 3.1. Diagnostic Approach

The first diagnostic tool is chest X-ray, which may show an elevation of the affected hemidiaphragm or both [5,7].

A notable finding in the diagnosis of the unilateral DP is a right hemidiaphragm elevation of two intercostal spaces or more above the left, or a left hemidiaphragm elevation of one intercostal space or more above the right [19].

In cases of bilateral diaphragmatic paralysis, diagnosis is more challenging because both hemidiaphragms appear elevated on X-rays images. In these cases, it is therefore necessary to perform dynamic tests that study their movement during the different respiratory phases.

Fluoroscopy provides a dynamic view of diaphragm excursion and must be performed with the patient breathing spontaneously. The exam can reveal paradoxical movement, hypokinesia, or akinesia [20].

In recent years, ultrasound has played an important diagnostic role. This exam is repeatable, can be performed bedside, and does not involve exposure to ionizing radiation. This technique has proven to be equivalent, if not superior, to fluoroscopy [21,22].

Kaur et al. recently described a case of diaphragmatic paralysis in a newborn, diagnosed and monitored by ultrasound, which made it possible to document the improvements, despite persistent requirement of respiratory support and avoid surgical plication [23].

Transcutaneous stimulation of the phrenic nerve combined with diaphragm electromyography allows for the evaluation of nerve conduction and motor response. These findings should be compared with the stimulation of the opposite phrenic nerve. The procedure can be carried out in patients on mechanical ventilation. Due to its high sensitivity and specificity, this technique is considered the gold standard for diagnosing DP [21].

CMAP obtained through transcutaneous stimulation of the phrenic nerves can be a prognostic tool to differentiate between prolonged PN recovery (neurapraxia with a likelihood of recovery) and the absence of signal (axonotmesis with no likelihood of recovery) [12,24].

Finally, brain MRI can be useful to exclude central causes of diaphragmatic paralysis, while cervical MRI can highlight potential damage to the nerve roots [20,25].

The PN originates bilaterally from the ventral branches of the vertebral nerve roots from C3 to C5 and are part of the cervical plexus. In the neck, they run along the ventral surface of the anterior scalene muscle, covered by the sternocleidomastoid. Both PNs pass through the thoracic inlet between the subclavian arteries and veins, cross the ipsilateral pleura, and begin their intrathoracic course [26,27,28,29].

The right PN runs laterally to the superior vena cava, the right atrium, the right ventricle, and the inferior vena cava, enters the vena cava foramen to access the abdominal cavity. The left PN descends laterally to the left subclavian artery, the aortic arch, the left atrial appendage, and the left ventricle before entering the abdominal cavity. The PNs branch out over their respective hemidiaphragms, providing the only motor innervation to the muscle [26,27,28,29].

It is therefore clear how nerve damage during cardiac surgery or complicated deliveries can lead to paralysis and eventration of the ipsilateral diaphragm, resulting in respiratory difficulties in the newborn.

Other, rarer causes, of DP include mechanical injury from the insertion of a chest tube or central venous catheter and the extravasation of parenteral nutrition around the phrenic nerves [29,30,31,32,33].

In birth trauma, the most common comorbidity associated with PN injury is BPP [34,35]. PN injury, when present, has been almost exclusively reported on the same side as BPP [7,10,36]. The only two anecdotal case reports of PN injury with contralateral BPP were described by Reiter et al. in 2020 and by Pegu et al. in 2018 [37,38].

However, in these cases, the DP affected only one hemidiaphragm, unlike our first case, where a perinatal injury from a difficult delivery led to bilateral involvement. To the best of our knowledge, this is the first case of its kind reported in the literature, posing a significant challenge in determining the cause and requiring us to rule out other possible explanations for the DP.

In the differential diagnosis of bilateral DP, it is mandatory to rule out genetic causes, which we excluded in the first case. The most common genetic causes are Spinal Muscular Atrophy with Respiratory Distress type 1 (SMARD1), Myotonic Dystrophy type 1 (MD1), Early-onset myopathy, areflexia, dysphagia, and respiratory distress (EMARD) and Myasthenia Gravis [39,40,41,42].

### 3.2. Therapeutic Approach

To date, there is no clear consensus regarding the optimal management of DP. In mild cases, careful monitoring and non-invasive respiratory support may be sufficient until spontaneous resolution of the injury [43].In more severe cases, invasive ventilatory support may be required until tracheostomy is performed [44]. Lastly, surgical dia-phragm plication could serve as an effective treatment for DP in cases with persistent, severe clinical symptoms [9].

However, given the variability in the likelihood and timing of spontaneous recovery, the ideal time for surgery remains an open question [45]. In both of our cases, the bilateral nature of the injury required an invasive intervention to allow weaning from mechanical ventilation. However, it was difficult to determine the timing and type of intervention, as there are no clear guidelines in the literature and because the recovery rate and time for such injuries are highly variable [7,12,36].

It is well known in the literature that the rate of spontaneous recovery following PN injury is lower in neonates who have experienced birth trauma compared to those who sustained the injury following cardiac surgery. Furthermore, the variability is extreme even within these two groups, with recovery occurring anywhere from a few months to several years after the injury [4,12,44].

A recent retrospective analysis of infants with unilateral diaphragmatic paralysis following cardiac surgery reported that conservative management with prolonged ventilation was effective in most cases. Nonetheless, the need for respiratory support beyond 21 days and a diaphragm elevation greater than two rib spaces were associated with a higher likelihood of requiring surgical plication [46].

Due to this extreme variability and the complications related to invasive mechanical ventilation, some authors suggest early diaphragmatic plication, within 20 days in neonates with obstetric trauma and within 10 days in those with post-surgical injuries [5,12].

Recent findings about DP secondary to phrenic nerve injury from birth trauma show that early surgical diaphragmatic plication, performed before 45 days of life, is associated with a shorter postoperative hospital stay, supporting early intervention as a favorable management strategy [47].

However, clinical studies evaluating the real efficacy of early plication compared to a more prolonged conservative approach are still lacking in the literature.

When, based on the patient’s clinical history, an invasive treatment is favored, the therapeutic options are essentially divided between tracheostomy and diaphragmatic plication. Experts recommend tracheostomy for bilateral DP to permit early oral intake and to accelerate the weaning process from ventilation [3,48]. These are the reasons why a tracheostomy was performed on our second patient at day 60 of life, with excellent respiratory outcomes.

Additionally, it is well known that the response to CPAP in patients with DP may predict the response to surgical plication [49]. Our second patient, once extubated and placed on non-invasive ventilation, exhibited immediate respiratory failure, with severe distress involving retractions, hypercapnia, and hypoxemia. Therefore, this patient was not considered a candidate for diaphragmatic plication.

The alternative to tracheostomy is diaphragmatic plication. Over time, the surgical approach to plication has changed. Historically, the access was via thoracotomy, but the procedure was associated with significant morbidity, such as severe scoliosis [50]. Nowadays, the thoracoscopic approach is preferred, particularly for DP involving the right hemidiaphragm [50,51]. Another option for repair is a laparoscopic approach for patients who may be more affected by a thoracic approach [52]. Therefore, the operative approach should be individualized based on the patient’s history and clinical characteristics, the laterality of the defect, and the experience and comfort level of the surgeon [53].

In our first patient, plication was postponed due to the extreme variability of spontaneous diaphragmatic function recovery and the parental desire to attempt a conservative approach. The final decision to proceed with surgery was made jointly with the surgical team and the parents, given the impossibility of weaning the child from invasive mechanical ventilation and thus proceeding with discharge.

To date, diaphragmatic pacing, used in the adult population in the treatment of respiratory failure associated with neurological dysfunction [54] is not an option for the neonatal population. To the best of our knowledge, only a few anecdotal attempts at diaphragmatic pacing have been described in patients with Central hypoventilation syndrome [55]. In the literature, pacing is not recommended in the pediatric population under nine months of age [56].

### 3.3. Our Clinical Challenges

The main challenges in managing our patients were identifying the etiology of diaphragmatic damage and selecting the appropriate therapeutic approach.

In the first case, the bilateral involvement of the PN, rarely associated with a dystocic delivery, and the difference in recovery time between the brachial plexus injury and the PN root lesion made it mandatory to rule out other underlying causes. Thus, an in-depth diagnostic process was necessary to identify potential alternative etiologies.

In the second case, the absence of other neurological symptoms in a patient who simultaneously presented other abnormal clinical and laboratory features suggested the possibility of diaphragmatic involvement as part of a complex, genetically indeterminate syndrome. This hypothesis directed the patient’s management in a broader context, where the diaphragmatic injury was not an isolated event but part of a multisystem condition requiring further investigation.

The significant variability in the rate and timing of diaphragmatic recovery, as extensively reported in the literature, made it difficult to make treatment decisions and provide families with an accurate prognosis.

In both cases, a conservative approach was initially adopted during the first month of life. However, this strategy was later changed to prevent complications associated with prolonged mechanical ventilation and to allow the families to take the patients home and continue their care there.

The decision to change the therapeutic approach was motivated by the need to improve the patients’ quality of life and by the desire to limit the negative effects of prolonged dependence on invasive ventilation, considering the individual clinical conditions and the preferences expressed by the families.

## 4. Conclusions

DP in neonates is a rare cause of respiratory distress and, as shown in our cases, determining the source of the damage is not always straightforward. Currently, there are no established guidelines for management, primarily due to the lack of robust data on the natural course of recovery. As a result, treatment must be tailored to prevent both acute and chronic respiratory issues and their potential complications.

## Figures and Tables

**Table 1:** Clinical features of the two patients.

	Patient n 1	Patient n 2
Sex	M	M
Gestational age	37 + 5	40 + 1
Mode of delivery	Operative delivery due to shoulder dystocia	Vaginal delivery
APGAR score	0, 3, 6 (perinatal asphyxia)	9, 10
Others features	Left humerus fracture BP involvment	Macrosomic, macrocephalic
Fluoroscopic evaluation	Inadequate bilateral diaphragm excursion	Reduced diaphragmatic excursion
Compound muscle action potential (CMAP)	Bilateral activity, with reduced left-sided response	Not performed
Electromyography (EMG)	Moderate peripheral neurogenic injury, affecting the C5–C6 roots on the right side, with signs of reinnervation but no active denervation	Normal EMG of the diaphragm
Magnetic Resonance Imaging (MRI)	Normal root courses of brachial plexus bilaterally	Normal MRI of the brain and spinal cord
Genetic	Exome analysis was negative	Paternal uniparental disomy (UPD) of chromosome 20 + A de novo heterozygous missense variant c.2983C > T in the SYNGAP1 gene
Treatment	Bilateral diaphragmatic plication at 45 days of age	Tracheostomy at 2 months of life
Follow up	Normal at 2 years, with physiotherapy and speech rehabilitation	Tracheostomy tube occluded at 2 years of life. Mild walking delay

## Data Availability

The original contributions presented in this study are included in the article. Further inquiries can be directed to the corresponding author.

## Abbreviations

The following abbreviations are used in this manuscript:

DP	Diaphragmatic paralysis
PN	Phrenic nerve
BP	Brachial plexus
BPP	Brachial plexus paralysis
CMAP	Compound muscle action potential
EMG	Electromyography
MRI	Magnetic Resonance Imaging
UPD	Uniparental disomy
NICU	Neonatal intensive care unit
EEG	Electroencephalogram
LGA	Large for gestational age
HFNC	High Flow Nasal Cannula
nCPAP	Nasal Continuous Positive Airway Pressure
LUS	Lung ultrasound
NGS	Next generation Sequency
SNP-array	Single nucleotide polymorphism-array
SMARD1	Spinal Muscular Atrophy with Respiratory Distress type 1
MD1	Myotonic Dystrophy type 1
EMARD	Early-onset myopathy, areflexia, dysphagia, and respiratory distress
